# In This Issue

**Published:** 1999

## WHAT IS MODERATE DRINKING?

In recent years, scientists and the media frequently have discussed the health benefits and risks associated with moderate alcohol consumption. To allow an informed debate, however, one must first define “moderate drinking” and, because many definitions of drinking levels are based on the consumption of a specific number of drinks during a certain time period, what constitutes a “drink.” Dr. Mary C. Dufour describes how the lack of a universally accepted definition of a drink may account in part for contradictory study results regarding the health consequences of moderate drinking. The differences in drink definitions and in the methodologies used to assess people’s alcohol consumption in turn lead to wide variation in definitions of “moderate drinking.” In the United States, nutritional guidelines of the U.S. Departments of Agriculture and of Health and Human Services define moderate drinking as no more than one drink per day for women and no more than two drinks per day for men. (pp. 5–14)

## MODERATE DRINKING AND REDUCED RISK OF HEART DISEASE

Coronary artery diseases (CADs), including heart attacks, account for approximately 25 percent of all deaths annually in the United States. The potential risks and benefits of moderate drinking on CAD have been widely discussed but often misinterpreted. Dr. Arthur L. Klatsky presents evidence indicating that moderate drinkers are at lower risk for CAD than are heavier drinkers or even abstainers. The association between moderate drinking and lower CAD risk may stem from health-related lifestyle factors (e.g., diet and exercise) shared by persons with similar drinking habits. In addition, some biochemical research supports a possible direct effect of alcohol itself. Among heavier drinkers, however, mortality risk increases significantly as drinking level increases, leading to unresolved questions about the balance between the risks and benefits of moderate drinking. (pp. 15–23)

## DRINKING MODERATELY AND PREGNANCY

Women who drink heavily during pregnancy run the risk of giving birth to children with a wide range of behavioral and developmental problems, the most severe of which is fetal alcohol syndrome. Children of women who drink moderately during pregnancy also may experience growth deficits and intellectual and behavioral problems similar to, although less severe than, those found in children with fetal alcohol syndrome. Drs. Joseph L. Jacobson and Sandra W. Jacobson describe the effects of moderate drinking during pregnancy on children’s development. They also examine the link between the child’s developmental problems and how much a woman drinks at one time and how often she drinks during pregnancy. (pp. 25–30)

## PREVENTING IMPAIRED DRIVING

A driver does not necessarily have to be intoxicated to be impaired by alcohol. Even moderate drinking can impair driving performance and increase the risk of a crash. Dr. Ralph W. Hingson, Dr. Timothy Heeren, and Mr. Michael R. Winter examine the relationship between alcohol consumption and impairment. They also summa-rizerecent research findings demonstrating how policy measures, such as legislated blood alcohol limits for drivers, are proving useful in reducing alcohol-related crash fatalities. (pp. 31–39)

## ALCOHOL AND MEDICATION INTERACTIONS

Combining even moderate amounts of alcohol with prescription or over-the-counter medications can prove dangerous. For example, as alcohol is broken down in the liver it may interfere with a medication’s metabolism, altering the way the drug is distributed in the body. Alcohol also may enhance the sedative effects of some medications, possibly impairing the drinker’s ability to perform certain tasks (e.g., driving a car). Drs. Ron Weathermon and David W. Crabb review the mechanisms that underlie such interactions. The authors also summarize the interactions between alcohol and various classes of medications. For example, alcohol metabolism in the liver may result in the accumulation of toxic by-products of some commonly used pain relievers. (pp. 40–54)

## GENDER DIFFERENCES IN MODERATE DRINKING EFFECTS

Women appear to be more susceptible than men to the intoxicating and adverse health consequences of alcohol consumption. Dr. Martin S. Mumenthaler and colleagues explore the basis of this finding, examining gender differences in the body’s processing of ingested alcohol and the brain’s sensitivity to alcohol’s physiological effects. The authors note that women reach higher blood alcohol concentrations than men after consuming equivalent amounts of alcohol, even when doses are adjusted for differences in body weight. In addition, women appear to be more susceptible than men to alcohol-induced impairment of the brain’s ability to process information, especially with tasks that require delayed memory or divided attention, skills important in driving a motor vehicle. Studies investigating potential effects of the menstrual cycle and variations in female sex hormones on women’s reactions to alcohol have been inconclusive. The data do suggest that women should not rely on non-gender-specific estimates of the amount of alcohol they can consume before becoming impaired. (pp. 55–64)

